# Future loss of Arctic sea-ice cover could drive a substantial decrease in California’s rainfall

**DOI:** 10.1038/s41467-017-01907-4

**Published:** 2017-12-05

**Authors:** Ivana Cvijanovic, Benjamin D. Santer, Céline Bonfils, Donald D. Lucas, John C. H. Chiang, Susan Zimmerman

**Affiliations:** 1 Climate Modeling and Analysis, Lawrence Livermore National Laboratory, 7000 East Avenue, Livermore, CA 94550-9698 USA; 2 Department of Geography and Berkeley Atmospheric Sciences Center, University of California, 547 McCone Hall, Berkeley, CA 94720-4740 USA; 3 Center for Accelerator Mass Spectrometry, Lawrence Livermore National Laboratory, 7000 East Avenue, Livermore, CA 94550-9698 USA

## Abstract

From 2012 to 2016, California experienced one of the worst droughts since the start of observational records. As in previous dry periods, precipitation-inducing winter storms were steered away from California by a persistent atmospheric ridging system in the North Pacific. Here we identify a new link between Arctic sea-ice loss and the North Pacific geopotential ridge development. In a two-step teleconnection, sea-ice changes lead to reorganization of tropical convection that in turn triggers an anticyclonic response over the North Pacific, resulting in significant drying over California. These findings suggest that the ability of climate models to accurately estimate future precipitation changes over California is also linked to the fidelity with which future sea-ice changes are simulated. We conclude that sea-ice loss of the magnitude expected in the next decades could substantially impact California’s precipitation, thus highlighting another mechanism by which human-caused climate change could exacerbate future California droughts.

## Abstract (web summary)

Persistent atmospheric ridging in the North Pacific steered storms away and led to the California drought of 2012-16. Here the authors use simulations to show that sea-ice changes trigger reorganization of tropical convection resulting in drying over California.

## Introduction

There is considerable uncertainty in model projections of twenty-first century precipitation changes over California^1, 2^. These uncertainties are largely a consequence of the sensitivity of Californian rainfall to both tropical and mid-latitude atmospheric circulation changes, such as variability in the El Niño/Southern Oscillation (ENSO) and shifts in the Pacific jet stream^2–4^. California’s winter precipitation has decreased over the past two decades, and between 2012 and 2016 California has entered into one of the most severe droughts on record^5–9^. The impacts of reduced rainfall have been intensified by anomalously high temperatures that have enhanced potential evapotranspiration^5^. Several studies posit that California’s drought has an anthropogenic component arising from increased temperatures, with the likelihood of such warming-enhanced droughts expected to increase in the future^5, 10, 11^.

The exceptionally dry conditions during the winters of 2012–2015 were accompanied by a prominent dynamical feature: a persistent geopotential ridge located in the North Pacific. This ridge pushed storm tracks further north, resulting in wetter than normal conditions over the northwest and substantial drying over the southwest of the United States^6^. A La Niña event in 2011/12 and anomalously warm sea-surface temperatures (SST) over the west tropical Pacific in 2012/13 and 2013/14 may have helped sustain the North Pacific geopotential ridge^6, 8^. Climate model simulations forced with observed SSTs alone were unable to capture all the important features of the recent precipitation decline, suggesting that California’s recent precipitation deficit may in part be attributable to other factors, such as internal atmospheric variability^8^.

A positive geopotential anomaly in the North Pacific is not unique to the recent drought. Similar anomalies are known to be associated with precipitation decline across the American southwest^4, 12^. Most of the existing literature points to anomalous tropical SSTs and convection as dynamical drivers of the geopotential changes in the North Pacific^6, 13, 14^. However, at least one study has implicated Arctic sea-ice loss as a possible cause of the drier winters over the American southwest^15–18^. One proposed physical mechanism involves surface warming and geopotential height increase over the newly open ocean areas in the Arctic, which in turn drives anomalous ridging over the North Pacific by perturbing high-latitude planetary wave patterns^15^. Although this mechanism is not considered to be a primary driver of California’s precipitation variability, scientific interest in the “sea-ice hypothesis” has been heightened by the pronounced Arctic sea-ice loss over the satellite era and the dramatic sea-ice retreat expected by the end of the twenty-first century^19^. Motivated by this, we focus on elucidating the possible mechanisms by which large-scale sea-ice cover changes could affect precipitation over California.

A novel feature of our approach is that we sample the uncertainties in selected sea-ice physics parameters (see “Methods” section and ref. ^20^), thereby obtaining an ensemble of simulations with a seasonally ice-free Arctic (“low Arctic ice” simulations). These are compared to control simulations representative of sea-ice conditions at the end of the twentieth century. A comparison between “low Arctic ice” and control simulations allows us to isolate the atmospheric response that is solely associated with changes in sea-ice cover. Neither the SSTs nor the sea-ice cover are prescribed in our model simulations. We are therefore able to account for atmosphere–surface ocean interactions and SST changes necessary for propagation of high-to-low latitude atmospheric teleconnections^21–23^. Unlike previous studies that impose artificial energy fluxes to achieve sea-ice loss^24, 25^, our sea-ice parameter perturbations allow for energy budget conservation.

Our primary interest is in the fast atmospheric response to sea-ice changes in the first decades after the sea-ice loss has occurred. This fast response is mediated by the surface ocean, and typically dominates the overall response during the first years or decades after the initial high-latitude perturbation^26–28^. We examine this initial response using an atmospheric general circulation model (AGCM) coupled to a mixed-layer ocean model. We do not investigate how this initial atmosphere–surface ocean response may become modulated by the slower deep-ocean response over longer (e.g., century scale) time periods as in refs. ^24, 25^. We verify that at the short timescales of interest here (<50 years after the sea-ice loss), an AGCM coupled to a slab ocean yields a qualitatively similar short-term response to that produced by the same AGCM coupled to a full ocean model (AOGCM) (see Supplementary Discussion). Our results show that the consequences of Arctic sea-ice decline are not restricted to the Arctic region alone. Loss of Arctic sea-ice cover of the magnitude expected in the next few decades, could induce large-scale atmospheric circulation changes across the Northern hemisphere, resulting in significant drying over California.

## Results

### Impacts of Arctic sea-ice loss on California’s precipitation

**Fig. 1:**
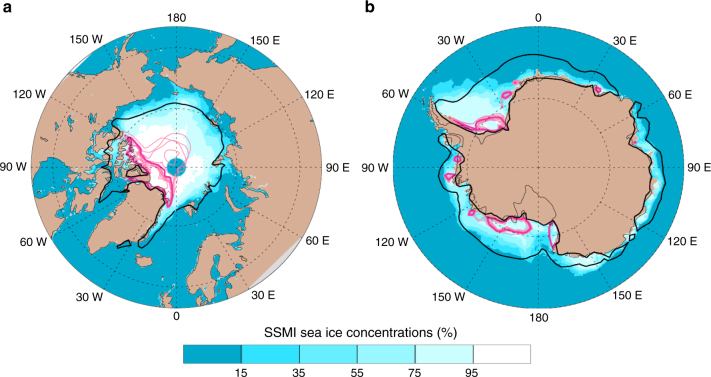
Impact of sea-ice physics parameter perturbations on Arctic and Antarctic sea-ice cover. **a** Arctic sea-ice concentrations, September. **b** Antarctic sea-ice concentrations, March. Shown are monthly mean sea-ice concentrations. Areas contained within contour lines have sea-ice fractions larger than 15%. Thick black lines denote control ensemble means, thick purple lines show “low Arctic ice” in **a** and “low Antarctic ice” ensemble means in **b**. Thin purple lines indicate individual “low Arctic ice” (“low Antarctic ice”) simulations. The colored shading indicates the average observed September (**a**) and March (**b**) sea-ice concentrations over the period 1992–2001 (from the Center for Satellite Exploitation and Research; http://nsidc.org/data/sipn/data-sets.html#seaice-conc-ext). A brief comparison of Arctic vs. Antarctic sea-ice changes is provided in Supplementary Note 1

Figure 1 and Supplementary Fig. 1 illustrate some of the differences in Arctic sea-ice cover between control and “low Arctic ice” simulations. During August and September, monthly mean Arctic sea-ice area for the “low Arctic ice” ensemble mean falls below the 1 million km^2^ threshold used to define a nearly ice-free Arctic^19^. By comparison, CMIP-5 simulations of twenty-first century climate and extrapolations of observed changes in ice volume place the time horizon for a nearly ice-free Arctic roughly between 2020 and 2040^19^. The smallest and largest differences between the “low Arctic ice” and control Arctic sea-ice area (Supplementary Fig. 1) occur in March and September, respectively, in accord with the seasonality of the observed Arctic sea-ice loss since the beginning of the satellite era.

**Fig. 2:**
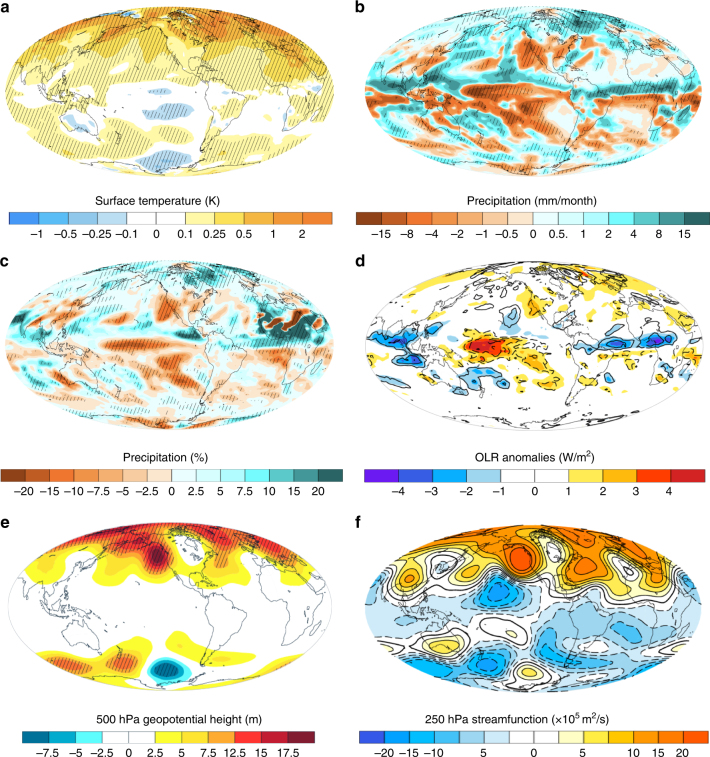
Atmospheric impacts of Arctic sea-ice loss. Shown are the ensemble mean differences (“low Arctic ice” minus control) for December–February (DJF) season. Stippling indicates anomalies that are statistically significant at the 90% confidence level. **a** Surface temperature anomalies. **b** Precipitation anomalies (absolute). **c** Precipitation anomalies (relative). **d** Outgoing longwave radiation (OLR) anomalies (shading) and the high cloud cover anomalies (contours). **e** 500 hPa geopotential distribution changes. **f** Stream function changes. Negative cloud cover anomalies in **d** are indicated with dashed lines

While the temperature impacts of sea-ice loss are most pronounced in the northern high- to mid-latitudes (Fig. 2a), precipitation anomalies show a more global response (Fig. 2b, c). We focus our analysis on the December–February season, because these months yield the largest impact of sea-ice changes on Californian precipitation in our model simulations. The most striking feature of the precipitation response to Arctic sea-ice loss is the reorganization of tropical rainfall and an apparent northward precipitation shift. The Arctic sea-ice decline also results in significantly less precipitation over California—a consequence of a geopotential ridge in the North Pacific that steers the wet winter air masses northward into Alaska and Canada, away from California (Fig. 2e).

These findings are consistent with previous claims of a sea-ice driven component of Californian precipitation changes^15, 29, 30^. We suggest, however, that the persistent geopotential height anomaly in the North Pacific shown in Fig. 2e is not just a direct consequence of sea-ice-induced surface warming and associated high-latitude planetary wave perturbations (as hypothesized previously), but rather is forced by sea-ice-induced convection changes over the tropical Pacific. We find that the key components of this two-step teleconnection are changes in the location and intensity of tropical Pacific convection and the northward-propagating Rossby wavetrain they initiate.

### Equatorward propagation

In the first step of this teleconnection, sea-ice loss alters the high-latitude energy budget (see annual mean flux changes shown in Supplementary Figs. 2 and 3a). These high-latitude energy flux changes are dominated by a decrease in the net top-of-atmosphere (TOA) radiation to space (Supplementary Fig. 2a). Sea-ice loss leads to an increase in the net downward TOA shortwave flux (Supplementary Fig. 2b) that is only partly compensated by an increase in net TOA upward longwave flux. This yields an increased net TOA heat flux into the atmosphere, and thus less radiation to space. Over the high northern latitudes, the TOA net flux changes are on average larger than the net surface flux changes (Supplementary Figs. 2a, c and 3a), leading to an increased heat flux into the atmospheric column (Supplementary Fig. 2d). With the exception of very high northern latitudes, sea-ice induced decrease in the net upward shortwave flux at the surface is largely compensated by an increase in the latent and sensible heat fluxes (Supplementary Fig. 2e, f, g). Several previous studies^30, 31^ support our finding of larger high-latitude TOA flux anomalies (relative to surface flux anomalies) in response to sea-ice changes. Experimental configurations that impose artificial surface energy fluxes in order to remove sea-ice cover necessarily yield a substantially larger surface energy imbalance than the one described here.

As a consequence of the increased high-latitude heat flux into the atmospheric column, the atmospheric heat transport from mid-latitudes into the high northern latitudes decreases (see the large white arrow in Supplementary Fig. 3a). The high-latitude energy surplus is compensated for at lower latitudes, with most of the energy emitted to space through the TOA flux changes over the tropical Pacific between 20**°**S and 20**°**N (Supplementary Fig. 3a, b). Increase in the tropical net TOA flux to space is associated with changes in high cloud cover. As seen from Supplementary Fig. 3b, regions of increased TOA flux to space coincide with the regions of decreased high cloud cover. An increase in the net TOA flux to space over the regions of decreased high cloud cover is a consequence of an increase in the outgoing longwave radiation (Supplementary Fig. 3c) due to decreased radiation height, that is only partially compensated by an increase in the net downward shortwave flux.

Tropical compensation of high-latitude energy budget perturbations has been noted in other studies employing AGCMs coupled to a slab ocean model^21, 23^. Such compensation is also evident in the short-term response to sea-ice changes in simulations employing fully coupled AOGCMs^30^. Specifically, previous work suggests that reorganization of atmospheric convection over the tropical Pacific (and resulting adjustment of the amount of energy emitted to space) can provide a compensation for energy budget perturbations elsewhere^21, 23^. This is not dissimilar to the observed changes in tropical TOA fluxes during ENSO-related convection changes that are also capable of affecting the global energy budget^32^.

The mechanism by which high-latitude changes propagate into the tropics and induce tropical convection responses involves two processes: (i) a southward advection of the initial high-latitude temperature perturbation over the mid-latitudes through transport and mixing; and (ii) a wind/evaporation/sea-surface temperature feedback in the region of the northeasterly trades. The tropical response to high-latitude sea-ice loss in our model simulations is consistent with these findings (see Supplementary Fig. 4). Once advected into the mid-latitudes, the sea-surface temperature (SST) anomalies propagate into the tropics by giving rise to anomalous meridional surface pressure gradients that in turn affect the strengths of easterly winds. These wind strength changes then further drive the evaporative flux changes^21-23^. Specifically, over the northern tropics, in the region of the northeasterly trades, the progression of cold (warm) SST anomalies will lead to an increase (decrease) in the surface wind strengths. This results in increased (decreased) evaporative cooling, thus allowing for further propagation of cold (warm) anomalies toward the equator. Over the southern tropics, the response is the opposite, due to the background southeasterly flow. Negative (positive) wind strength and latent heat flux anomalies over the northern (southern) tropics in response to Arctic sea-ice loss are shown in Supplementary Fig. 4b, c.

The progression of these relatively small SST changes into the tropics, in combination with tropospheric temperature changes aloft, can drive convection changes in the tropical Pacific^21-23^. Areas of large zonal and meridional temperature gradients adjacent to deep convection regions in the tropical Pacific represent so called “convective margins”^23, 33, 34^. These are areas where small changes in atmospheric conditions can initiate or halt deep convection. Even weak sea-surface temperature anomalies (Supplementary Fig. 4a), in combination with small tropospheric temperature changes (Supplementary Fig. 4d), are sufficient to trigger convection changes over these regions (Fig. 2d).

### Poleward propagation

We suggest that the tropical Pacific convection changes described above play a key role in driving the North Pacific circulation changes. Linkages between the equatorial Pacific convection anomalies and extratropical (North Pacific) atmospheric circulation changes are found in both observational and modeling studies^13, 35, 36^ and have been invoked to explain North Pacific circulation differences during different phases of the ENSO cycle. These studies find that enhanced winter convection (and corresponding enhanced upper-level divergence) in the equatorial Pacific initiates a northward-propagating Rossby wavetrain. The characteristics of this wavetrain are positive stream function and geopotential anomalies immediately outside the region of increased convection (anticyclonic flow), and negative anomalies in the extratropical Pacific (cyclonic flow). We find that this mechanism also operates in our simulations.

During the DJF season, the area of strongest decrease in high cloud cover is located over the central tropical Pacific (see contour lines in Fig. 2d). This region of decreased tropical deep convection coincides with the area of the largest outgoing longwave radiation (OLR) increase (see shading in Fig. 2d). In the second teleconnection step, decreased deep convection in the tropical Pacific (Fig. 2d) and the associated decrease in upper-level divergence force a northward-propagating Rossby wavetrain with negative (cyclonic) anomalies in the northern tropical Pacific and positive (anticyclonic) anomalies in the extratropical North Pacific sector. This is evident from the 250 mb stream function changes (Fig. 2f), which indicate anomalous cyclonic flow in the tropics and anomalous anticyclonic flow in the North Pacific. The 250 mb North Pacific anticyclonic flow is the upper-level manifestation of the geopotential ridge in Fig. 2e—a factor responsible for steering the wet winter air masses away from California. The atmospheric anomalies in the North Pacific exhibit an equivalent barotropic response (Supplementary Fig. 5a).

**Fig. 3:**
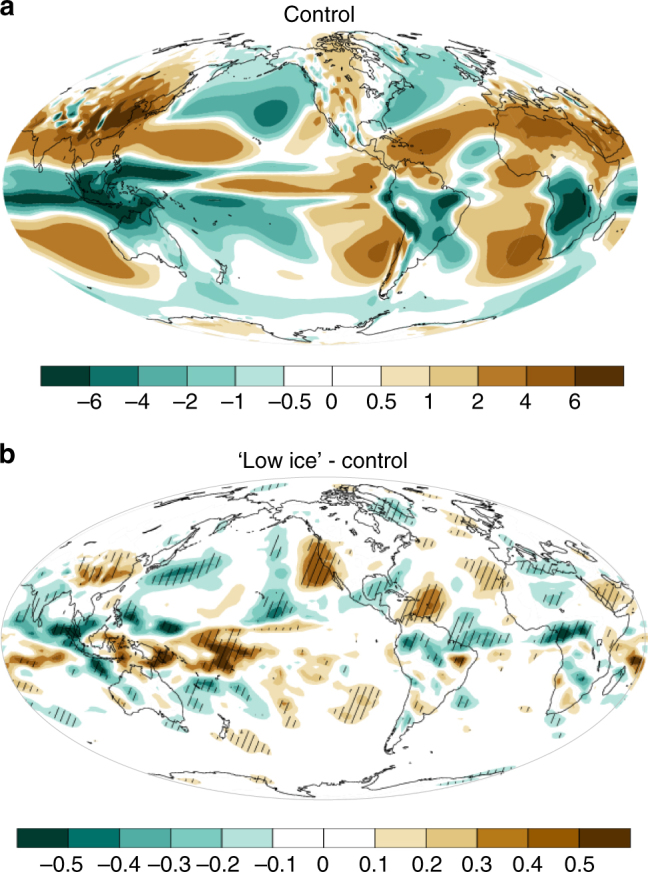
Changes to 250 mb vertical velocity (hPa/s). **a** Control simulation climatology. **b** The response to Arctic sea-ice loss (“low Arctic ice” minus control ensemble mean). Positive sign denotes subsidence. Stippling in **b** indicates anomalies that are statistically significant at the 90% confidence level

The convection changes described above are consistent with the simulated upper-level vertical velocity anomalies and Hadley circulation changes. From a climatological perspective, the descending branch of the winter Hadley cell is positioned over (and to the west of) California (Fig. 3a and Supplementary Fig. 6). Arctic sea-ice loss markedly amplifies this subsidence over California, while also affecting the uplift and subsidence regions across the tropical Pacific (Fig. 3b). The tropical Hadley circulation plays a key role in the atmospheric transport of heat between the tropics and extratropics^21, 37, 38^. Changes to the Hadley circulation are an expected and known dynamical response to the energy budget perturbations shown in Supplementary Fig. 3 ^21-23, 39^.

**Fig. 4:**
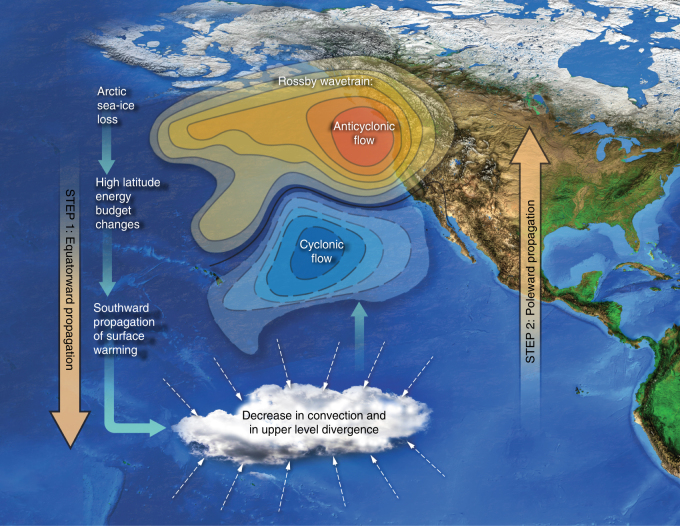
Schematics of the two-step teleconnection. In step 1 (equatorward propagation), Arctic sea-ice loss induced high-latitude changes propagate into tropics, triggering tropical circulation and convection response. Decreased convection and decreased upper-level divergence in the tropical Pacific in turn drive a northward-propagating Rossby wavetrain with anticyclonic flow forming in the North Pacific. This ridge is responsible for steering the wet tropical air masses away from California

The schematic in Fig. 4 summarizes the hypothesized two-step teleconnection, involving sea-ice loss, tropical convection reorganization, a northward-propagating Rossby wavetrain, and a decrease in precipitation over California. Several previous studies have suggested that tropical and subtropical Pacific convection changes affect precipitation over California by inducing geopotential anomalies in the North Pacific^6, 8, 13^. While such tropical and subtropical convection changes could be a consequence of natural variability, our analysis indicates that they can also be forced from the high latitudes by substantial sea-ice loss. Our simulations imply that two hitherto separate hypotheses—that Californian rainfall is primarily influenced by tropical convection changes (“tropical hypothesis”)^6, 7, 11^ and that Arctic sea-ice loss can drive precipitation changes over California (“Arctic sea-ice loss hypothesis”)^15, 29, 30^—are not easily separable. Our study demonstrates that the impacts of Arctic sea-ice changes require understanding complex atmospheric teleconnections that can propagate back and forth between the extratropics and the tropics.

### Atmospheric impacts of Antarctic sea-ice loss

**Fig. 5:**
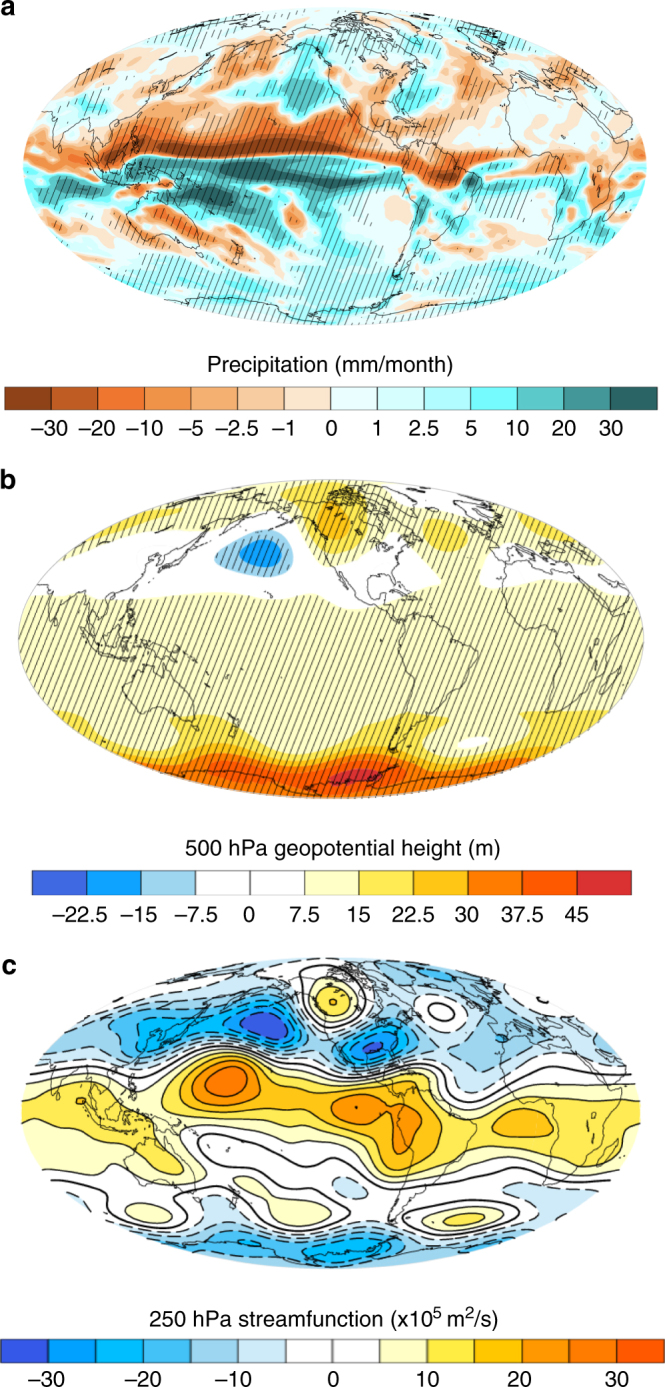
Atmospheric impacts of Antarctic sea-ice loss. **a** Precipitation anomalies. **b** 500 hPa geopotential distribution changes. **c** Stream function changes. Shown are the ensemble mean differences (“low Arctic ice” minus control) for December–February (DJF) season. Stippling indicates anomalies that are statistically significant at the 90% confidence level

To further investigate the proposed two-step teleconnection, and additionally verify that sea-ice-induced tropical convection changes are forcing the circulation changes in the North Pacific, we explore the impacts of another source of remote forcing: Antarctic sea-ice loss. Antarctic sea-ice decline will result in a warming at mid- to high latitudes of the southern hemisphere. One consequence of this warming is a southward shift of tropical precipitation, with convection increase in the southern and central tropical Pacific^40^. This is the opposite to the precipitation response accompanying Arctic sea-ice loss. In accord with previous studies^13, 35, 36^ and the mechanism advanced here, increased convection should in turn drive a northward-propagating Rossby wavetrain consisting of an anticyclonic anomaly in the tropical Pacific and a cyclonic anomaly in the North Pacific. This cyclonic flow is associated with negative geopotential height anomalies over the North Pacific (geopotential low) leading to wetter conditions over California. Simulations in which the sea-ice physics parameter perturbations are prescribed in the Southern hemisphere only (“low Antarctic ice” simulations, Fig. 1b and Supplementary Fig. 1b, d) show a response that is consistent with these expectations. Tropical precipitation shifts southward (Fig. 5a), anticyclonic flow is established over the northern tropical Pacific, and cyclonic flow is formed over the North Pacific (Fig. 5c). The geopotential ridge in the North Pacific is replaced with a geopotential trough, favoring the propagation of tropical storms across California (Fig. 5b). As a result, precipitation over California increases (Fig. 5a).

The “low Antarctic ice” simulations show that the proposed mechanism can be triggered by sea-ice changes in either hemisphere. Since Antarctic sea-ice loss involves northward propagation in both teleconnection steps (i.e., Antarctic sea-ice affecting the tropical Pacific, which in turn affects the North Pacific) and no high northern latitude changes, it provides additional support for our conjecture that the sea-ice changes can influence North Pacific geopotential height through tropical convection changes. More generally, any high-latitude perturbation (northern or southern hemispheric warming or cooling) that impacts the position of the tropical Pacific ITCZ, will have an impact on California’s rainfall.

## Discussion

Many aspects of the observed mean state of the atmosphere during the recent 2012–2016 California drought are qualitatively consistent with our simulated response to Arctic sea-ice changes. ERA-Interim estimates of the precipitation, geopotential, and OLR anomalies between 2012 and 2015 (relative to the period 1979–2010) are shown in Supplementary Fig. 7. Similar to our simulations, ERA-Interim exhibits a geopotential ridge in the North Pacific and a precipitation decrease over California (Supplementary Fig. 7a, b). In the tropics, precipitation decreases across the central and eastern tropical Pacific and increases northward of this region and in the west tropical Pacific (Supplementary Fig. 7a). Another commonality is the behavior of OLR changes across the central tropical Pacific and over California (see Supplementary Fig. 7c). The magnitudes of the observed 3-year mean anomalies during the recent California drought (Supplementary Fig. 7a) are much larger than the magnitudes of the 20-year mean response to sea-ice changes shown in Fig. 2a. This is expected given that the longer 20-year period contains both dry and wet years. As seen from Supplementary Fig. 8a, low Arctic sea-ice increases the likelihood of drier California, but does not result in drier conditions over California every single year. On average, when considering the 20-year mean, there is a 10–15% decrease in California’s rainfall (Fig. 2c). Comparison with the driest 3-year interval within this 20-year period (Supplementary Fig. 8b) indicates that the magnitude of the simulated precipitation response is comparable to the magnitude of changes in ERA-Interim during the most recent drought.

This consistency does not, however, constitute compelling evidence that the 2012–2016 California drought is attributable to Arctic sea-ice changes. Rather, it illustrates that some of the atmospheric features of the droughts driven by Arctic sea-ice loss may resemble those of the most recent California drought. The intensification of dry conditions over California since late 2012 may have also been affected by several other factors not discussed in this study, such as the appearance of a large warm SST anomaly off the west coast of North America—though this appears to be a consequence rather than a cause of the altered atmospheric circulations causing the drought^41^. Another important factor may be the 2014 phase shift of the Pacific Decadal Oscillation from negative to positive. However, this shift would be expected to alleviate drought conditions over California—not intensify them^42^. Finally, we note that other hemispherically asymmetric forcings may also trigger—via the same physical pathways described here—tropical convection changes and teleconnections affecting California’s precipitation. Possible examples include asymmetric forcing by anthropogenic aerosols, volcanic eruptions, and solar irradiance variations. All of these forcings can alter the inter-hemispheric temperature gradient^43^, thereby causing shifts in the location of tropical convection and initiating “far-field” precipitation changes over California.

It is of interest to consider whether paleoclimatic evidence provides support for our model-inferred two-step teleconnection (Fig. 4). We focus on past periods with pronounced excursions in Arctic temperatures and sea-ice cover. The first part of the two-step teleconnection is well documented in both paleoclimate simulations and paleoclimate records of the last glacial period^44^. Abrupt high-latitude temperature increase during the last interglacial (the Dansgaard–Oeschger and Bølling–Allerød warmings) are manifest in the tropics through a northward shift in the position of the ITCZ^45–47^. Conversely, the signature of cold stadials and Younger Dryas cooling in the Arctic is matched by a southward shift of tropical precipitation across all ocean basins^45–48^. These ITCZ shifts are consistent with precipitation changes inferred from high-resolution, precisely dated speleothem records: speleothems across the southwestern United States indicate shifts to drier conditions during the Dansgaard–Oeschger warmings^49, 50^, the Bølling–Allerød warming, and the termination of the Younger Dryas cooling^51^. Similarly, during the Heinrich 1 and Younger Dryas cold periods, wetter conditions are indicated by lake-level high stands in the Great Basin and glaciation in central and southern California^52, 53^, while at least one modeling study has implicated North Atlantic cooling and a corresponding southward ITCZ shift as a possible cause of increased precipitation over the western United States^54^.

The timing, magnitude, and drivers of sea-ice changes during the last millennium are less well understood^55^. In addition, volcanic activity may have also had an impact on tropical precipitation during this time^56^. However, proxy data for the last millennium does yield information regarding the second teleconnection step—the link between the ITCZ location and southwestern rainfall. The southward shift of the ITCZ in the transition from the medieval climate anomaly (MCA) to the little ice age (LIA) is well documented^57, 58^. The effect of this shift is apparent in the contrast between the dry MCA and the wet LIA in the western US lake levels^59^, from trees growing on lake beds exposed by low lake levels^60, 61^, and in pollen records^62^. Interestingly, a brief southward shift in the ITCZ position between ~AD 1050 and 1250^57^ is clearly recorded in the American southwest as a wet interval in the midst of the dry MCA. Great Basin lakes expanded briefly during this period^63, 64^, concurrent with drowning of trees growing below modern water levels^60, 61^ and increased soil moisture in southern California (as inferred from pollen records)^62^. Such periods during the MCA and LIA provide some support for the second teleconnection step. In the absence of more reliable sea-ice data sets, however, we cannot confirm if such covariance relationships between the ITCZ location and southwestern rainfall are directly attributable to sea-ice changes, or could instead be due to other factors (e.g., volcanic forcing or solar irradiance changes).

Finally, we address the suitability of our experimental configuration (an AGCM coupled to a slab ocean) for studying the fast atmosphere–surface ocean-mediated response to sea-ice changes. In our simulations, key response features are manifest within the first two decades (see Supplementary Fig. 9). While our current study does not examine how this initial atmosphere–surface ocean response may be altered on longer timescales by the deep ocean, we note that the fast response identified here is in agreement with previous AOGCM-based studies^15, 29, 30^. Additional analysis of the short-term (first 50 years) response of an AOGCM response to sea-ice changes in a complementary, energy-conserving setup confirms our AGCM/slab ocean findings (see Supplementary Discussions and Supplementary Fig. 10). The decadal-scale AOGCM response indicates tropical convection changes and drying over California in response to the loss of Arctic sea-ice cover, a qualitatively similar result to that seen in the present study. Recent studies have investigated the impacts of Arctic sea-ice loss in a fully coupled AOGCM setup by imposing artificial high-latitude energy fluxes^24, 25^. These studies have demonstrated the importance of ocean dynamics one century after imposing high-latitude longwave forcing. However, such investigations provide no evidence that ocean dynamics dominates the response to high-latitude longwave forcing on timescales considerably shorter than a century, nor do they indicate when the transition to an ocean-dominated response would occur. Understanding the differences between energy-conserving and non-energy-conserving setups, in particular how the presumed impacts of sea-ice changes in non-energy-conserving experimental setups^24, 25^ may be affected by artificially imposed energy flux perturbations remains an important future step. A key result from our study is the identification of a link between high-latitude and tropical energy budget and convection changes that can further drive precipitation changes over California. This link highlights the importance of differentiating between: (i) the different timescales of the response; and (ii) studies that attempt to isolate the impacts of sea-ice changes by imposing large artificial energy perturbations in order to achieve the sea-ice loss^24, 25^, and investigations that achieve sea-ice changes in an energy-conserving manner (ref. ^30^ and the current study).

Selection, implementation, and testing of sea-ice physics parameter changes from the latin hypercube simulation ensemble^20^ has provided a novel energy-conserving framework for testing the climate impacts of sea-ice changes. Our results implicate both Arctic and Antarctic sea-ice loss as potential drivers of future precipitation changes over the American southwest. We show that substantial loss of high-latitude sea-ice cover is likely to have significant far-field effects, and can impact California’s precipitation through atmospheric teleconnections involving tropical convection changes.

The present day precipitation decreases over California and the associated circulation anomalies are not evident in coupled model projections of twenty-first century climate^65^. Most current simulations disagree regarding the sign of the future precipitation changes over California^65^. As a consequence, a multi-model mean features a large envelope of uncertainty, and suggests negligible precipitation decline in the first decade of the twenty-first century, followed by an increase in precipitation^8, 65^ over the next decade. One possible cause of these inter-model discrepancies is that some climate models underestimate the magnitude of observed Arctic sea-ice loss over the satellite era^19^ and the sign of recent Antarctic sea-ice changes^66, 67^. Our analysis suggests that both of these factors—the reduced magnitude of Arctic sea-ice loss and the reversed sign of Antarctic sea-ice changes relative to the observations—contribute to the smaller than observed precipitation decline in model simulations of early twenty-first century climate change. Our results imply that as long as Arctic sea-ice cover continues to decrease, overall sea-ice forcing will favor drying over California. This assumes that Antarctic sea-ice continues to expand or remains stationary over the next several decades. As Antarctic sea-ice cover begins to decrease, the competing influences of Arctic and Antarctic sea-ice loss will weaken or even reverse the sign of the sea-ice driven component of California’s precipitation changes. We emphasize, however, that sea-ice loss is one of multiple factors implicated in driving the described atmospheric circulation and precipitation changes. In simulations of historical and future climate, multiple hemispherically asymmetric forcings are changing simultaneously. Some of these forcings affect sea-ice cover, thus hampering the elucidation of links between Arctic sea-ice loss and California’s rainfall. This is one advantage of the experimental configuration used here. By design, the convection, atmospheric circulation, and precipitation responses described here are directly related to sea-ice changes in each hemisphere, and not to changes in anthropogenic and natural forcings, or deep-ocean feedbacks.

We have confirmed that the short-term (decadal-scale) response to Arctic sea-ice loss described in our study is consistent with the response found in previous AOGCM studies that allow for ocean dynamics and deep-ocean changes. The long-term centennial climate response to sea-ice changes may, however, differ from the fast response described here. While investigations with other climate models are necessary to further confirm these findings, our results strongly suggest that what happens in the Arctic does not stay in the Arctic and provide a physically plausible pathway by which high-latitude sea-ice forcing may mediate tropical climate, and thereby influence California’s rainfall.

As a final remark, we note that the pronounced Arctic sea-ice loss over the satellite era is likely human-induced, arising from anthropogenic warming caused by greenhouse gas increases^68^. Our study thus identifies yet another pathway by which human activities could affect the occurrence of future droughts over California—through human-induced Arctic sea-ice decline.

## Methods

### Model

We use the Community Climate System Model (CCSM) version 4^69^. The model configuration incorporates the Community Atmosphere Model version 4 (CAM4)^70^, Community Land Model version 4 (CLM4)^71^, and Los Alamos Sea Ice Model version 4 (Community Ice CodeE 4—CICE4)^72^ coupled to a mixed-layer (slab) ocean. The atmosphere and land models are run on a 1.9° × 2.5° (latitude × longitude) finite volume grid with 26 atmospheric levels in the vertical dimension. The ice and ocean models use a 1° displaced pole grid. The greenhouse gas concentrations, solar insolation, and orbital parameters are representative of the year 2000 values for all simulations. The prescribed ocean heat flux (“q-flux”), salinity, temperature, and velocity fields required for the mixed-layer ocean model simulations are derived from a fully coupled CCSM simulation using the full-depth ocean.

### Perturbed sea-ice physics parameter simulations

In order to isolate the atmospheric impacts of sea-ice loss, we compare two sets of experiments that differ only in the amount of sea-ice cover. For this purpose, we have developed a novel experimental configuration in which we alter the sea-ice cover without imposing artificial energy fluxes in the high latitudes as done in the previous studies. When sea-ice loss is achieved by imposing non-physical heat flux perturbations, energy is not conserved. This makes it difficult to determine if the observed atmospheric response originates solely from the sea-ice changes or if it is also impacted by the imposed energy flux perturbations. Analyzed response represents cumulative effect of sea-ice extent and thickness changes.

Sea-ice loss in our model simulations is achieved through sea-ice physics parameter perturbations. We use an ensemble of 70 Latin hypercube simulations to select the sea-ice physics parameters that have the strongest impact on the resulting sea-ice extent^20^. The selected sea-ice physics parameters are shown in Supplementary Table 1. Parameter values were not varied outside of their respective expert-defined ranges. Two sets of sea-ice physics parameter values are chosen, both resulting in similar amounts of sea-ice loss (see Supplementary Fig. 1 and “Methods” section). Parameter perturbations are separately applied in each hemisphere: “low Arctic ice” (“low Antarctic ice”) simulation sets are obtained by applying the parameter changes in the Northern (Southern) hemisphere alone. For each set of ice parameter values used (control, set 1 and set 2) we performed five additional simulations with altered initial atmospheric conditions. The length of each model simulation was 40 years. Our focus is on the last 20 years of these simulations. “Low Arctic ice” and “low Antarctic ice” ensemble means are an average of all simulations with the perturbed sea-ice parameter values applied in the Northern and Southern hemispheres, respectively. The anomalies represent the ensemble mean differences between “low Arctic ice” (or “low Antarctic ice”) and control simulations.

Statistical significance is estimated using Welch’s *t*-test in order to account for different sample size and variances between the control and “low Arctic ice” simulations. Prior to *t*-test calculations, the equivalent sample size is determined at each grid point (accounting for temporal autocorrelation).

### Data availability

Simulation output processed for the purpose of this study has been made public and can be obtained at https://esgf-node.llnl.gov/projects/psipps/. Provided are monthly means of the following CAM4 variables: ice fraction (ICEFRAC), surface pressure (PS), surface temperature (TS), upper tropospheric temperature (TROP_T), total precipitation (PRECT), geopotential (Z3), horizontal and meridional wind speed components (U and V), vertical (pressure) velocity (OMEGA), net longwave flux at the atmosphere top (FLNT), and high cloud cover (CLDHGH); all for years 20–40 of model integration. Additional data can be made available on request.

Perturbed sea-ice physics parameter simulations require changes to the following parameters: snow grain radius tuning parameter, thermal conductivity of snow, and snow melt maximum radius. Their respective values are given in Supplementary Table 1. Implementation of the perturbed sea-ice parameter values requires namelist and CICE4 source code changes (available per request).

## Electronic supplementary material

Peer Review File

Supplementary Information
