# Supplementary material for: Future loss of Arctic sea-ice cover could drive a substantial decrease in California’s rainfall
Source: Nat Commun. 2017 Dec 5;8:1947. doi: 10.1038/s41467-017-01907-4 (PMC5717256; doi:10.1038/s41467-017-01907-4)
Supplement: Supplementary file 2 — Supplementary Information [file 41467_2017_1907_MOESM2_ESM.pdf]

Supplementary Information

Cvijanovic et al.: Future loss of Arctic sea-ice cover could drive a substantial decrease in California's rainfall

Includes: Supplementary Discussion, Supplementary Note 1, Supplementary Table 1, Supplementary Figures 1-10, Supplementary References 1-6.

Supplementary Discussion

We test whether the mechanism advanced for our slab ocean AGCM simulations is robust to the inclusion of ocean dynamics. For this purpose, we use a set of previously published, fully-coupled AOGCM simulations (1), in which the sea-ice changes are induced in an energy-conserving manner, but through modification of ocean albedo values rather than as perturbations to sea-ice physics parameters. The atmospheric component of the AOGCM model is the same as that used in our AGCM/slab ocean runs. The presence of a deep ocean response is not the only difference between the slab ocean AGCM setup used in our study and the AOGCM simulations described in ref. (1). Nevertheless, such a comparison is more appropriate than a comparison with other existing simulations that feature fresh-water forcing (2) or artificial energy flux perturbations (3, 4). Here, we investigate whether the AOGCM simulations qualitatively capture the basic features of the teleconnection between Arctic sea-ice changes, tropical Pacific precipitation and convection reorganization, and precipitation changes over California. We do not attempt to make detailed comparisons regarding the magnitude or exact location of changes. As in our AGCM/slab ocean analysis, we focus on short-term changes, commencing a couple of decades after the initial sea-ice perturbations establish.

Precipitation, OLR and geopotential changes arising from Arctic sea-ice loss in AOGCM simulations (1) are shown in Supplementary Fig. 10. The anomalies are an average between years 20 to 50 of fully-coupled model integration in an experiment in which ocean albedo perturbations are imposed between 75-90° N. The annual mean difference between the control and perturbed total sea-ice area is ~1.46 million km^2. In agreement with our AGCM analysis, precipitation anomalies (Supplementary Fig. 10a) indicate that in a low state of Arctic sea-ice cover, California is substantially drier compared to the corresponding high sea-ice state. Large precipitation anomalies are also present in the tropical and subtropical Pacific. Substantial precipitation increase (decrease) is present over the northeast (southwest) tropical Pacific. OLR anomalies from the AOGCM simulations reveal large positive anomalies over the west tropical Pacific (decreased deep convection) and negative anomalies over the northeast tropical Pacific (increased deep convection) (Supplementary Fig. 10b). The geopotential anomalies indicate the presence of a geopotential ridge that extends across the entire North Pacific (Supplementary Fig. 10c). These AOGCM inferred anomalies suggest that AOGCM simulations capture the basic characteristics of the AGCM/slab ocean response.

The primary differences between the results from the AGCM/slab ocean and the AOGCM are as follows: a) the AOGCM simulation shows a substantially larger precipitation response; b) AOGCM tropical precipitation changes are less zonal; c) areas of large positive OLR anomalies and North Pacific geopotential and precipitation changes are shifted somewhat westward in the

AOGCM; and d) poleward of 70°N, 500 hPa geopotential changes show different features. In the AOGCM simulation, deep convection decreases over the western tropical Pacific and the geopotential ridge spreads across most of the North Pacific, while the precipitation decrease is centered over the eastern subtropical Pacific, reaching California with its eastward flank. Given the overall experimental differences between the AOGCM and the AGCM/slab ocean simulations (different complexities of ocean model components, initial conditions, greenhouse gas concentrations, and methods of inducing sea-ice changes), it is not possible to attribute these differences in the magnitudes and exact geographical locations of the responses to a specific cause (e.g., deep ocean changes). One interesting topic for future research is the role of increased latent heat transport due to higher CO_2 concentrations in fully coupled simulations.

In summary, we find that the AOGCM simulations reproduce many of the same basic features of the response to sea-ice changes obtained in our AGCM/slab ocean configuration. This suggests that the response mechanisms we have identified in the simpler AGCM/slab ocean experiments are physically reasonable. Other altered albedo simulations in ref. (1) also support this conclusion.

Supplementary Note 1

In our model simulations, sea-ice physics parameter changes have different impacts on Artic relative to Antarctic sea-ice loss. In ‘low Arctic ice’ simulations, imposed parameter changes result in the seasonally ice-free Arctic: the ensemble mean September sea-ice area is ca. 0.5 million km^2 (compared to 4.5 million km^2 in the control) (Fig. 1). Parameter changes imposed in the Northern Hemisphere lead to the strongest sea-ice loss in September and the weakest in March (Supplementary Fig. 1a). In the Antarctic (Supplementary Fig. 1b), there is no substantial change in sea-ice area between the ‘low Arctic ice’ and control simulations (total ice area is about 14 million km^2 in both). This is expected since the parameter changes were imposed in the Northern Hemisphere only.

In ‘low Antarctic ice’ simulations (Supplementary Fig. 1, panels c and d), parameter changes are imposed in the Southern hemisphere only. This results in substantial Antarctic sea-ice loss, larger than the respective Arctic sea-ice loss seen in ‘low Arctic ice’ simulations. ‘Low Antarctic ice’ simulations also have less pronounced variation in the amplitude of the seasonal sea-ice loss than it was the case in the ‘low Arctic ice’ simulations. As a consequence, December-February sea-ice loss is larger in ‘low Antarctic ice’ than in the ‘low Arctic ice’ simulations. The stronger response of Antarctic sea-ice cover to imposed parameter changes partly explains the larger atmospheric responses to Antarctic sea-ice loss in our model simulations (relative to Arctic sea-ice loss case).

Imposed parameter changes do not only affect the sea-ice area, but also the sea-ice thickness. Similar to the sea-ice area changes, thickness changes are also larger in ‘low Antarctic ice’ than in the ‘low Arctic ice’ simulations. The studied response is a consequence of both of these factors – sea-ice thickness and area changes.

Supplementary Tables

Supplementary Table 1: Sea-ice physics parameter ranges and values

Parameter	expert defined range	Control	‘low Arctic ice’ set 1	‘low Arctic ice’ set 2
snow grain radius tuning parameter	-1.9 to 1.9	1.5	-1.8	-0.84
thermal conductivity of snow	0.1 to 0.35	0.3	0.1	0.1
snow melt maximum radius	500 to 2000	1500	1800	1362.7

Supplementary Figures

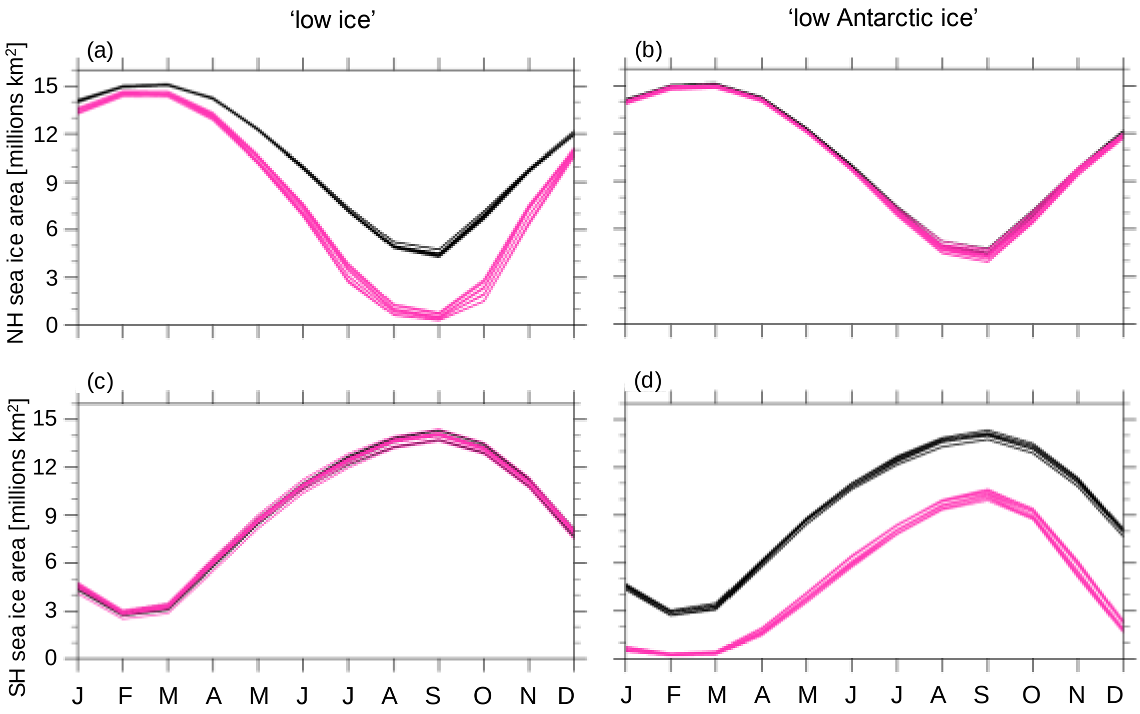

Supplementary Fig. 1: Hemispheric averages of monthly mean sea-ice areas. Purple lines indicate 'low Arctic ice' (a,c) and 'low Antarctic ice' (b,d) simulations. (a,b): Northern and (c,d): Southern hemispheric means. Black lines show the corresponding sea-ice areas in 'control' simulations. A brief comparison of Arctic versus Antarctic sea-ice changes is given in Supplementary Note 1.

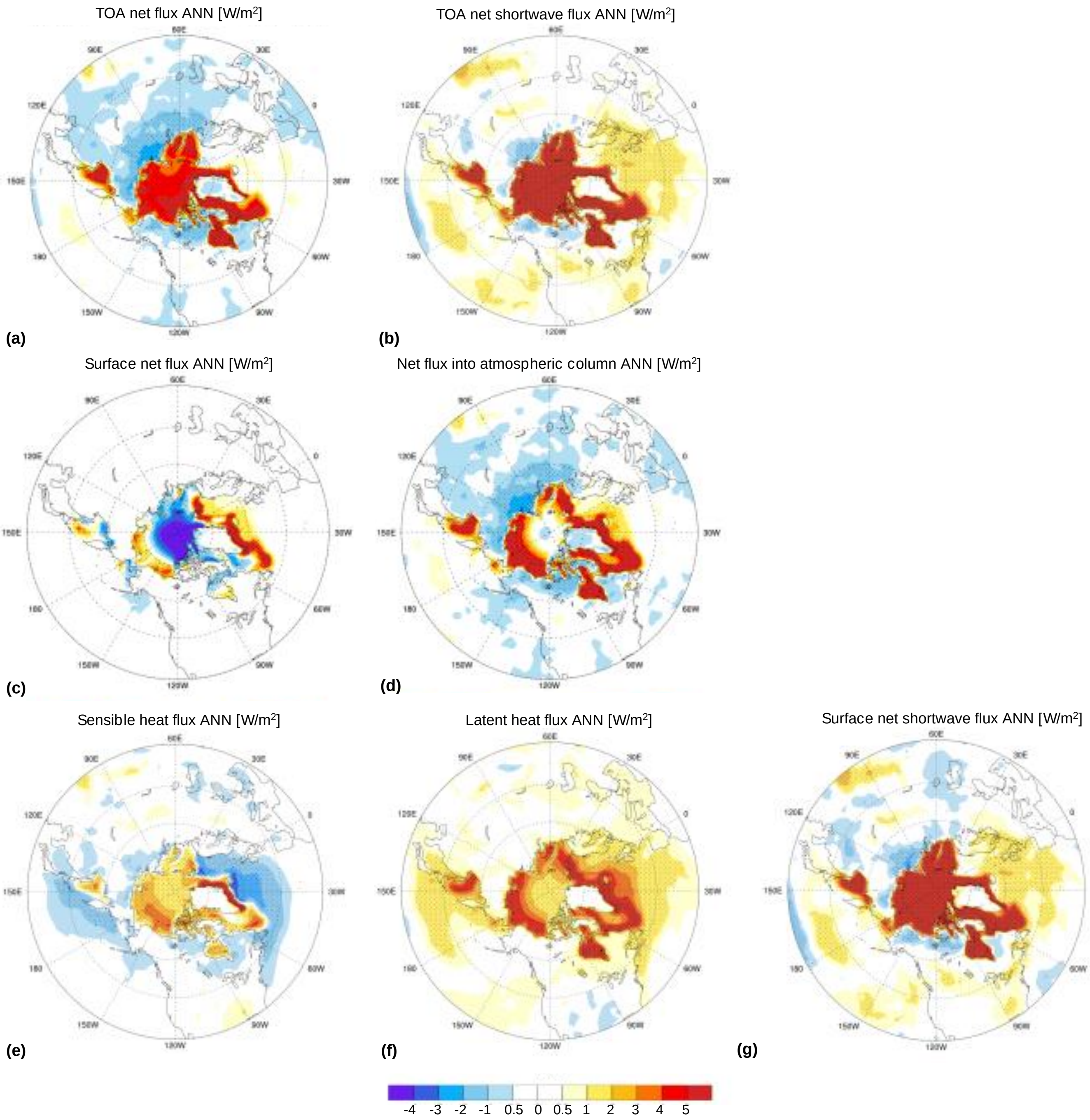

Supplementary Fig. 2: High-latitude annual mean net energy flux changes due to Arctic sea-ice loss. ‘Low Arctic ice’ minus control ensemble means. Shown are: net top-of-atmosphere (TOA) heat flux (positive down) (a); TOA net shortwave heat flux (positive down) (b); net surface heat flux (positive up) (c); net heat flux into the atmosphere column (TOA net – surface net) (d); sensible heat flux (positive up) (e); latent heat flux (positive up) (f); and net downward shortwave flux at the surface (g).

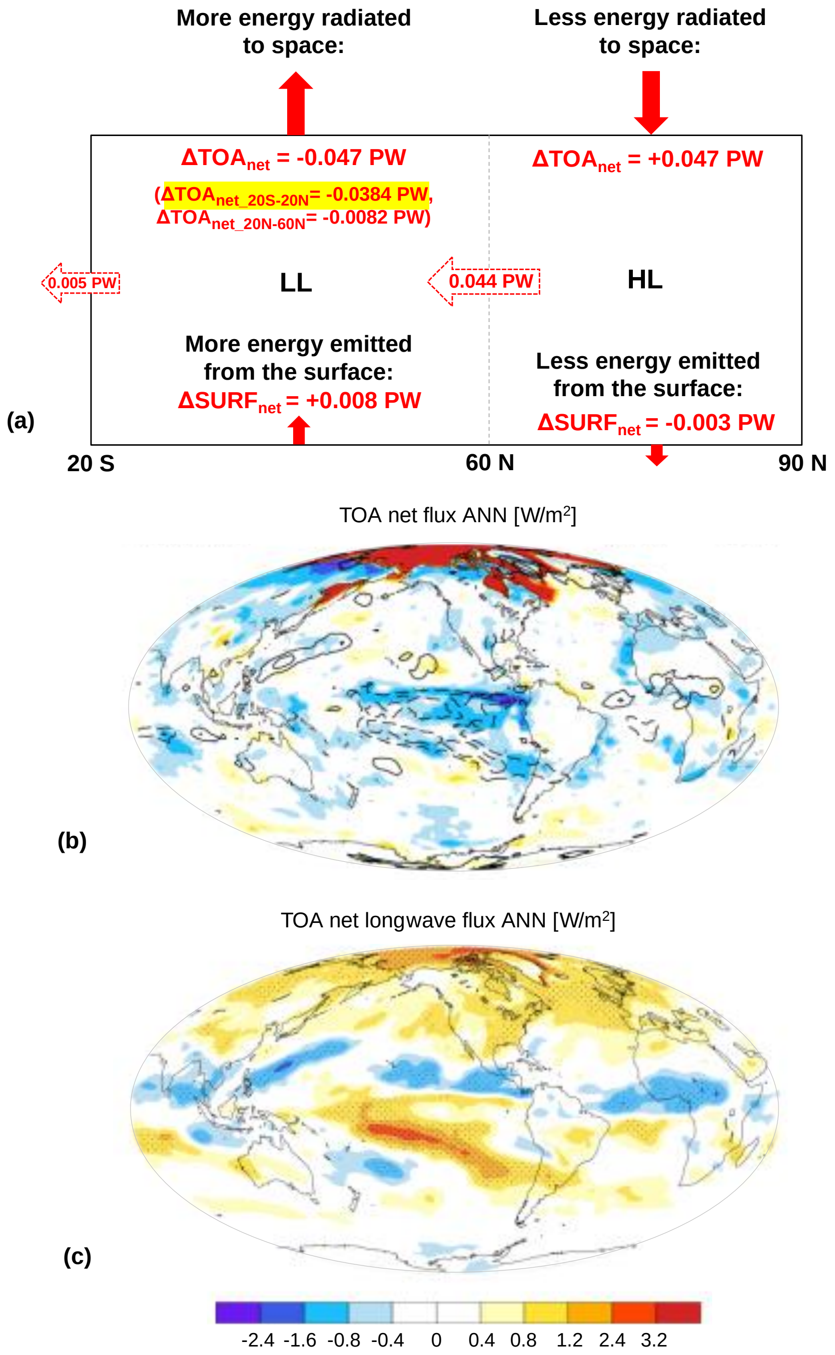

Supplementary Fig. 3: Global annual mean net energy flux changes due to Arctic sea-ice loss. 'Low Arctic ice' – control ensemble means. (a) The schematic showing high-latitude energy budget changes between 20°S and 90°N. Red arrows denote relative values of surface and TOA flux changes. White arrows indicate implied changes in atmospheric heat transport. (b) TOA net heat flux anomalies (positive down). (c) Outgoing longwave radiation (OLR) anomalies (c). Contour lines in panel (b) indicate high cloud cover changes (negative changes - dashed).

Over the high latitudes (HL, 60°N - 90°N) sea-ice changes alter both surface and TOA energy fluxes with annual mean TOA changes being substantially larger (0.047 PW compared to -0.003PW). This high-latitude energy surplus is compensated for in the lower latitudes (LL) with most of the energy (0.038 PW) being emitted back to space over the area 20°S and 20°N.

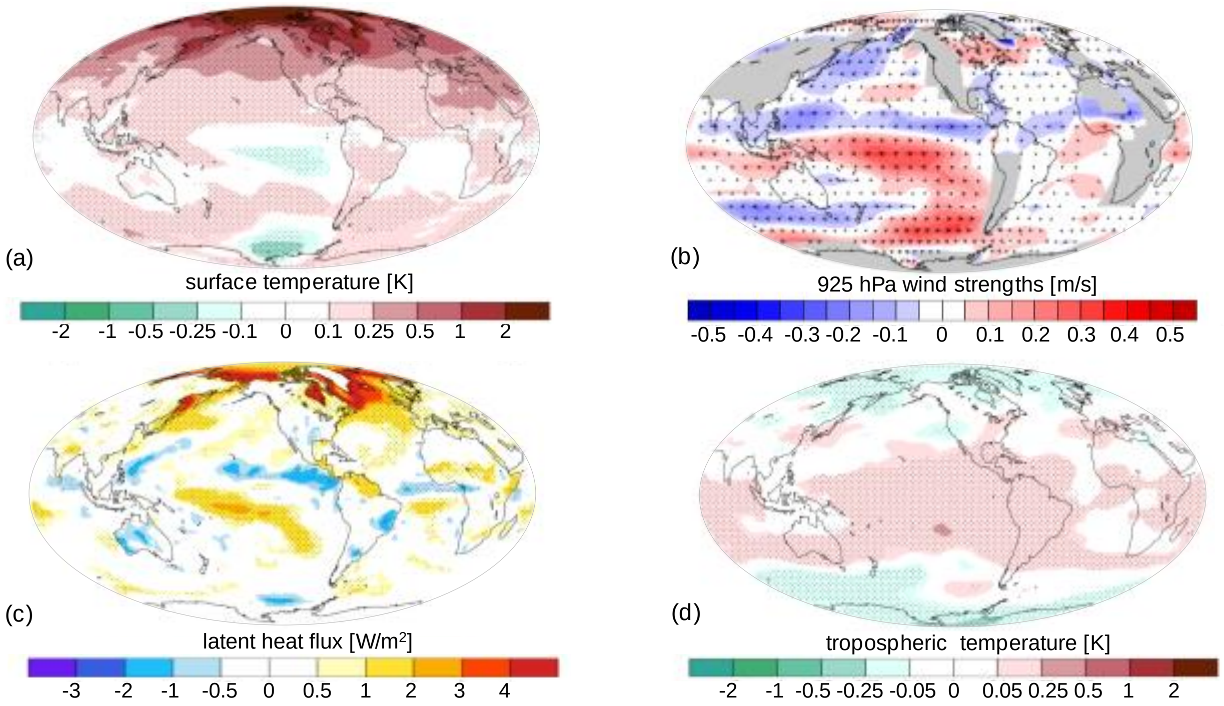

Supplementary Fig. 4: Atmospheric impacts of Arctic sea-ice loss. Shown are annual mean anomalies ('low Arctic ice' – control ensemble means). (a) Surface temperatures. (b) Surface wind strengths. (c) Latent heat flux. (d) Tropospheric temperatures. Small dots in panels a, c and d indicates anomalies that are statistically significant at the 90% confidence level.

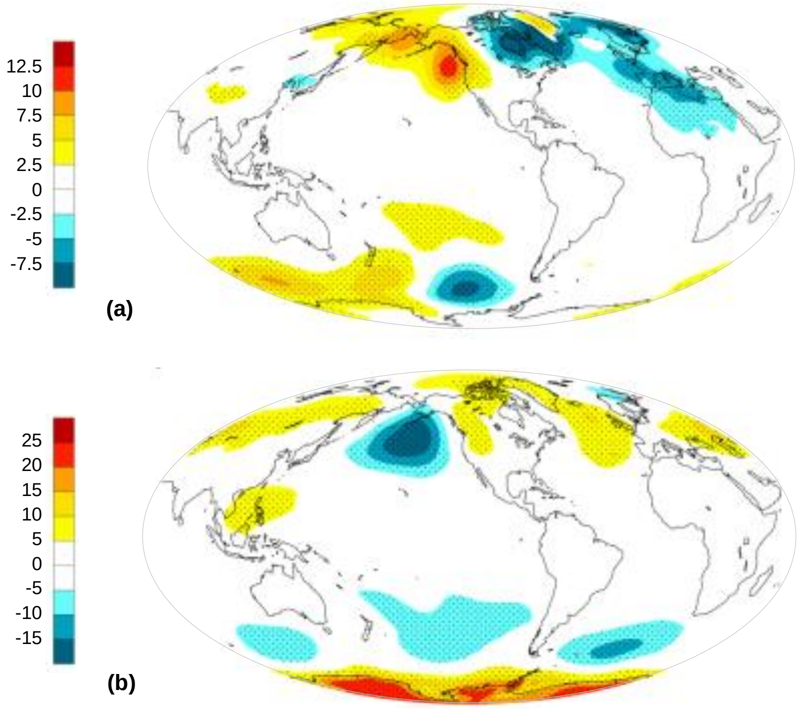

Supplementary Fig. 5: Impacts of Arctic and Antarctic sea-ice loss DJF surface pressure anomalies. (a) ‘Low Arctic ice’ simulations. (b) ‘Low Antarctic ice’ simulations. Positive (negative) surface pressure anomalies over the North Pacific in panels (a) and (b) coincide with the positive (negative) geopotential anomalies aloft (Figs.2e and 5b), indicating an equivalent barotropic response to Arctic and Antarctic sea-ice forcing. Small dots denote anomalies that are statistically significant at the 90% confidence level.

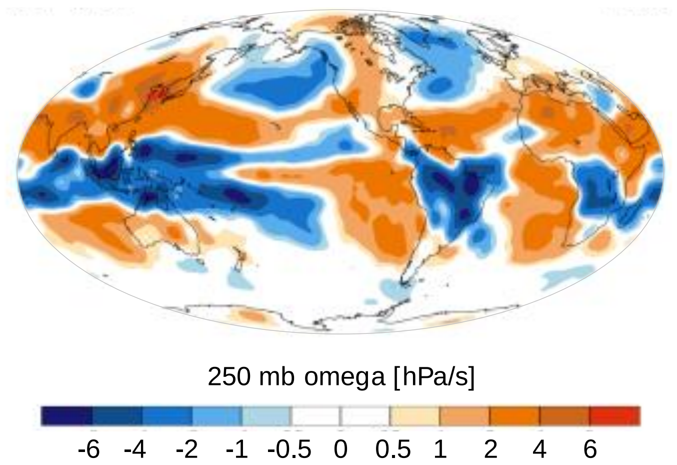

Supplementary Fig. 6: NCEP 250 mb vertical velocity. Winter (DJF) mean from 1980 to 2000. From NCEP reanalysis (5), data provided by Physical Sciences Division, Earth System Research Laboratory, NOAA, http://www.esrl.noaa.gov/psd/. Comparison with Fig. 3a shows that our model simulations successfully capture the upper level vertical velocity fields from the reanalysis data.

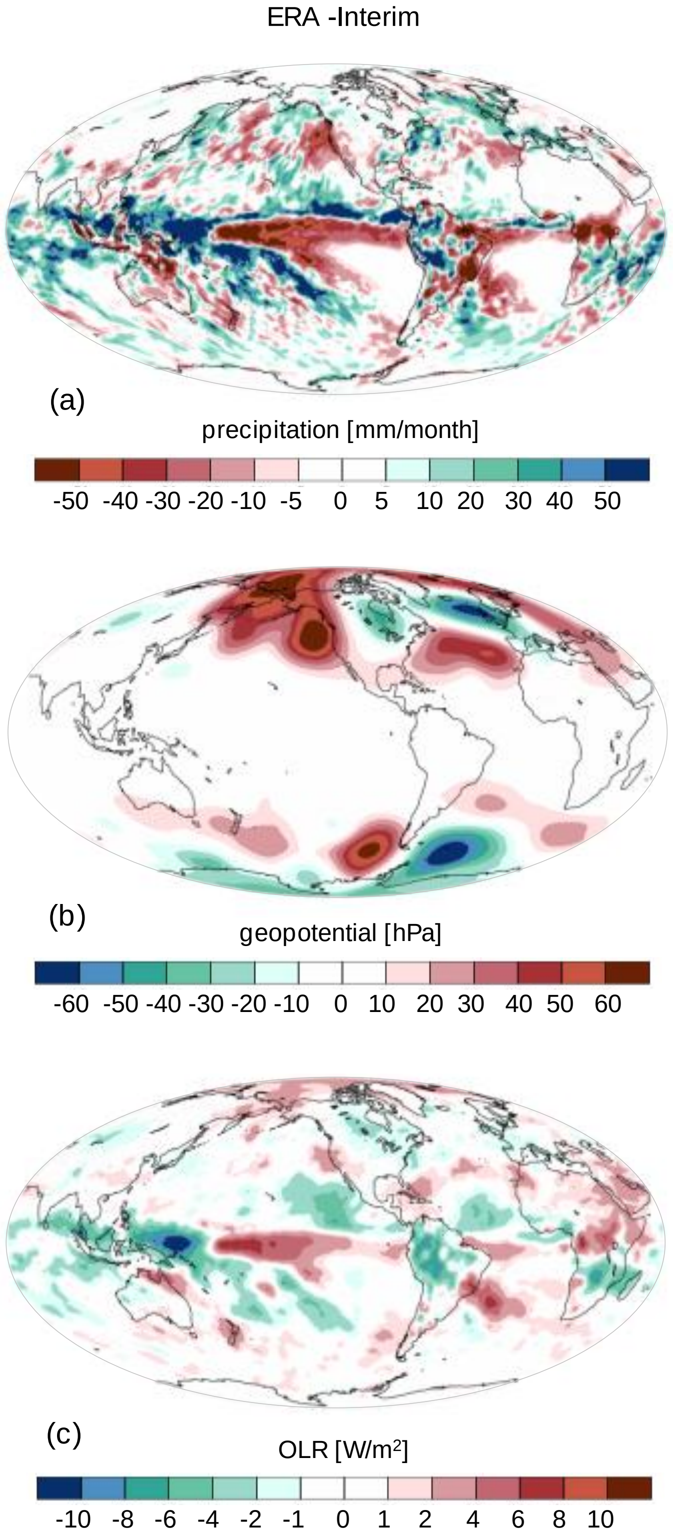

Supplementary Fig. 7: ERA-Interim DJF anomalies, 2012-2015 relative to 1979-2010. (a) precipitation, (b) geopotential and (c) Outgoing Longwave Radiation. Data source for panels a-c: European Center for Medium-Range Weather Forecast ERA-Interim reanalysis (6).

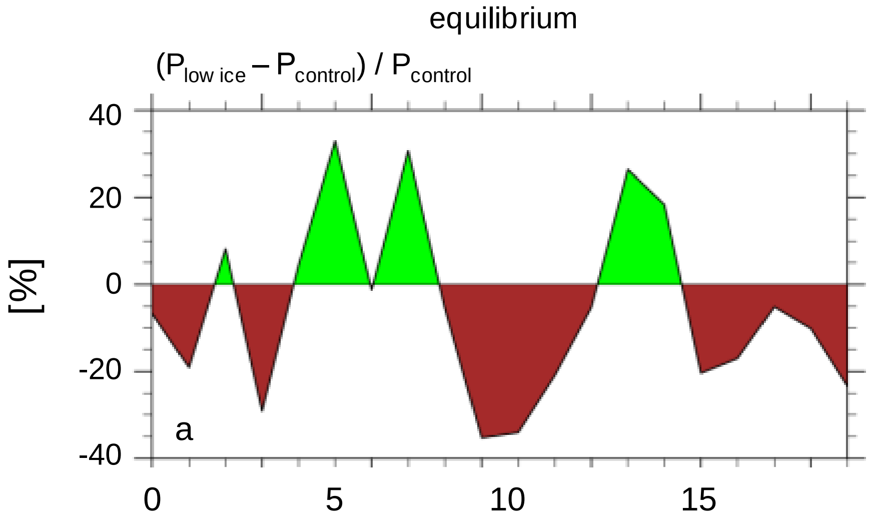

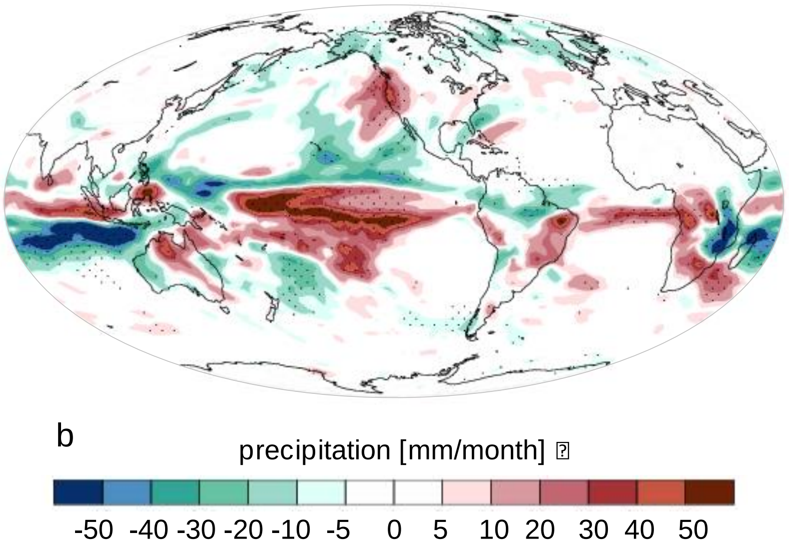

Supplementary Fig. 8: (a): Relative precipitation change [%], (‘low Arctic ice’ – control)/control, area averaged between 33 – 43 °N and 116 – 125 °W. Results are December – February ensemble-mean differences over the last 20 years (equilibrium) of model integration (20 years containing 19 DJF seasons). (b): Precipitation anomalies over the driest 3-year interval (‘low Arctic ice’ – control) within the equilibrium period (the last 20 years of model integration). Small dots in panel (b) indicate anomalies that are statistically significant at the 90% confidence level.

The large precipitation variability in the transient response is also present in the equilibrium response. While the precipitation anomalies shown in Fig. 2b and Supplementary Fig. 8b are both statistically significant (as indicated by the stippling and small black dots), large year-to-year precipitation variability over California (8a) results in climatological precipitation anomalies (20-year means) in Fig. 2b that are smaller than the anomalies during the driest 3-year period (8b).

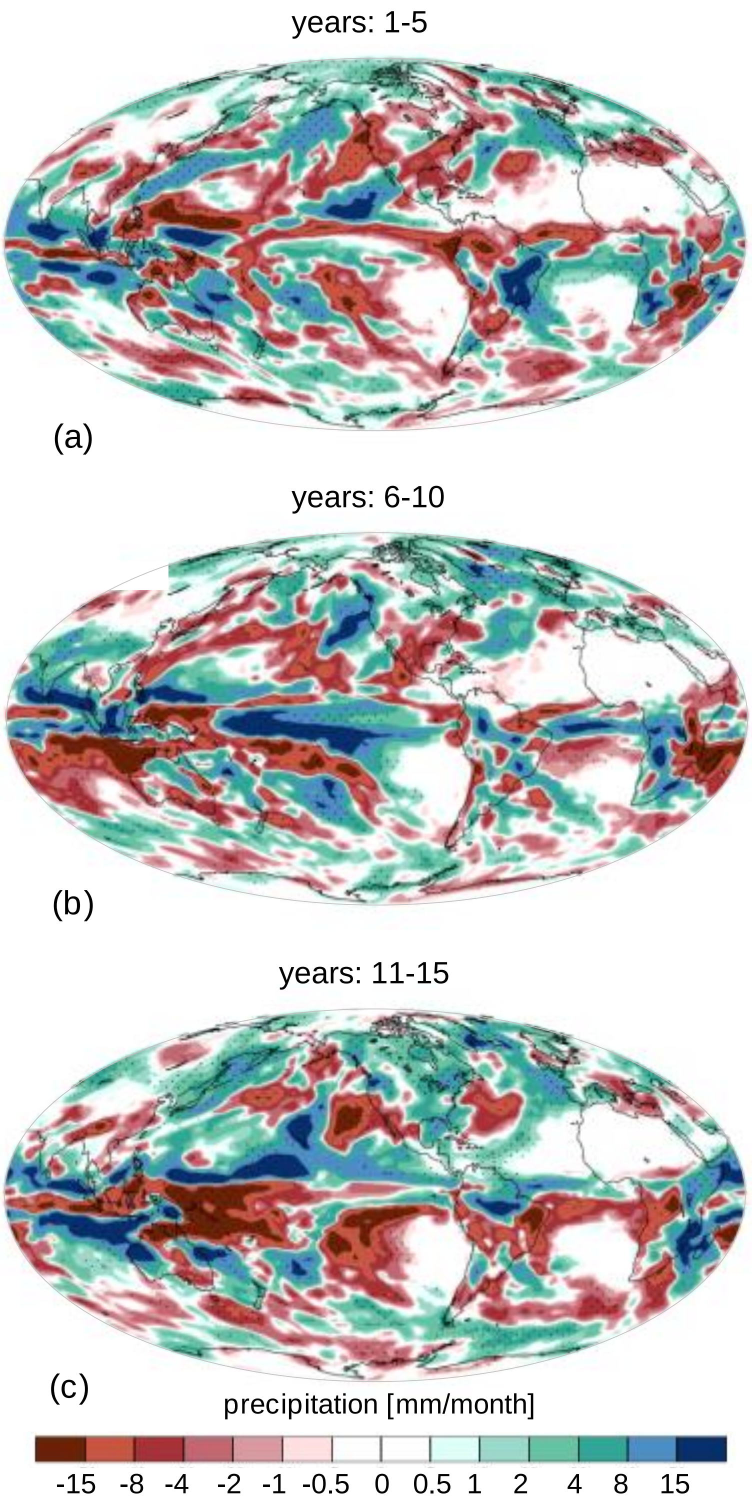

Supplementary Fig. 9: Transient response to Arctic sea-ice loss. Shown are December-February precipitation anomalies. Years: (a) 1-5. (b) 6-10. (c) 11-15. Small black dots indicate anomalies that are statistically significant at the 80% confidence level. During the first five years of model integration (a), the loss of Arctic sea-ice cover leads to a weak northward precipitation shift over the east tropical Pacific and some drying over California. During years 6-10 (b), the sign of the response changes, with a southward ITCZ shift in the Pacific and wetter conditions over California. After year 10, the main features of the equilibrium response are established (c).

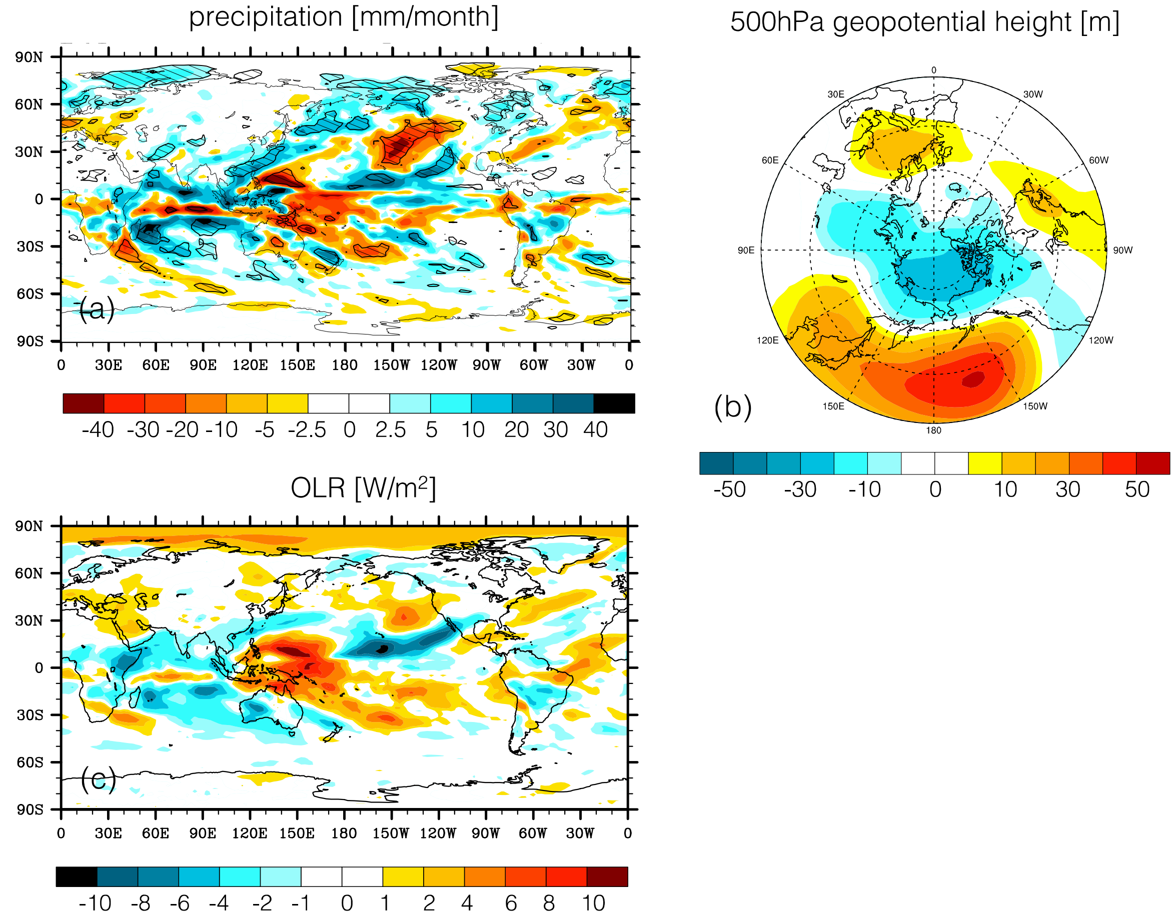

Supplementary Fig. 10: Winter (DJF) response to Arctic sea-ice changes from fully coupled simulations. (a) Precipitation anomalies. (b) 500 hPa geopotential anomalies. (c) Outgoing Longwave Radiation changes. Differences shown here are between the control (‘low Arctic ice’) and alb75-90 (‘high ice’) experiments (control minus alb75-90, see ref. 1 for more details). Dashed areas in (a) indicate anomalies that are statistically significant at the 90% confidence level.

Supplementary References:

1. Cvijanovic I, Caldeira K, MacMartin DG, Impacts of ocean albedo alteration on Arctic sea ice restoration and Northern Hemisphere climate. *Environ Res Lett* 10(4): 044020 (2015)
2. Zhang R, Delworth TL, Simulated Tropical Response to a Substantial Weakening of the Atlantic Thermohaline Circulation. *J Clim* 18(12):1853–1860 (2005).
3. Deser C, Tomas RA, Sun L, The Role of Ocean–Atmosphere Coupling in the Zonal-Mean Atmospheric Response to Arctic Sea Ice Loss. *J Clim* 28(6):2168–2186 (2015).
4. Deser C, Sun L, Tomas RA, Screen J, Does ocean coupling matter for the northern extratropical response to projected Arctic sea ice loss? *Geophys Res Lett* 43(5):2149–2157 (2016).
5. Kalnay et al. The NCEP/NCAR 40-year reanalysis project, Bull. Amer. Meteor. Soc.77: 437-470 (1996).
6. Dee DP et al. The ERA-Interim reanalysis: Configuration and performance of the data assimilation system. *Quart. J. Roy. Meteor. Soc*. 137: 553–597 (2011)
