# Supplementary material for: Future loss of Arctic sea-ice cover could drive a substantial decrease in California’s rainfall
Source: Nat Commun. 2017 Dec 5;8:1947. doi: 10.1038/s41467-017-01907-4 (PMC5717256; doi:10.1038/s41467-017-01907-4)
Supplement: Supplementary file 1 — Peer Review File [file 41467_2017_1907_MOESM1_ESM.pdf]

Reviewer #1 (Remarks to the Author):

see uploaded document

Reviewer #2 (Remarks to the Author):

In this contribution, Ivana Cvijanovic and her co-authors use simulations with an AGCM to establish a possible link between sea-ice changes in high latitudes and precipitation changes over California. In doing so, they in particular examine the physical mechanisms that might explain the linkages they find.

I very much enjoyed reading this paper, and found the results presented here both novel and convincing. This work will certainly inspire future studies and is thus in my opinion well suitable for publication in Nature Communications, subject to some rather minor changes as indicated below. I should note that I was particularly impressed by the authors' own capability to discuss the strengths and limits of their approach and to very clearly spell out possible uncertainties in their findings. Because of this, I don't have any substantial criticism regarding the scientific arguments brought forward here.

Minor comments:
l.55: Include possibly reference to Fig. S8b.

l.77: To me, the term "climate model" today usually refers to an AOGCM. I got confused when I later realised that this term is here meant to represent an AGCM, and think this should be made clear upfront to avoid such confusion.

l.80: I wasn't sure if the location of reference (18) is as intended. Doesn't this reference explain the simulations described in the previous sentence, rather than the comparison to the control simulation?

l.87ff: It never became clear to me why this study is not based on the results from the AOGCM that the authors refer to occasionally. While I appreciate that the results might not change qualitatively as discussed in S1.6, the robustness of the results would have been strengthened by using an AOGCM as opposed to an AGCM.

l.111: It would be nice to show SST also in Fig 2.

l.111-114: I found these remarks distracting within this paragraph. Can they be moved to the Methods?

l.114-116: Can be removed, as this was already described in l.111.

l.130ff: I found Supplemental Section 1.2 very helpful in understanding these findings. It would be good if at least some parts of the Supplemental Section could be incorporated into the main text, given its importance to understand the following reasoning.

l.241ff: I found the logical flow of the discussion not always clear, as it currently jumps several times between a comparison to observational data and possible limits of the present approach. Instead, one could start with a comparison to recent observational data (l.274-290), the final sentence of which could then nicely be followed by the paragraph in lines 268-273. Then, one could compare the present findings to paleo data, moving back in time. (i.e., first ll 255-267, then ll. 241 to 254). This would then end the comparison to earlier data. Then, the discussion starting in l. 291 could follow.

l.402: Some publications have strange additional notes in capital letters (e.g. 6, 10, 24, 61)

Figure 1: I recommend to replace Figure 1b with Figure S1e, and to change the caption accordingly. I'm not sure that it is necessary to include Fig. 1b and S1f. Instead, it can simply be stated that sea-ice coverage in the unperturbed hemisphere is very similar to the control simulation.

Text S1.6 and S1.7: The numbers for references in these two sections seem to be too low by 1. (e.g, ref. (26) should be (27) etc.)

Fig. S3e: The main text seems to imply that the dashed lines describe changes in clouds. However, I believe that this is not explained in the caption.

Response to reviewers' comments on "Seasonally ice-free Arctic favors dry California" by I. Cvijanovic, B. D. Santer, C. Bonfils, D. D. Lucas, J.C.H. Chiang and S. Zimmerman

Please accept our sincere apologies for the major delay in the delivery of this response. [redacted]

We hope that the topic discussed in our paper is still of interest to the Editorial Office of Nature Communications, despite the "hiatus" in the paper's revision.

The reviewers' comments were very useful and helpful – we are grateful to both reviewers for their constructive comments, thorough consideration and respectful communication. The requested changes have been implemented as described below. All line numbers pertain to the revised version of the paper.

Sincerely,

Ivana Cvijanovic (on behalf all the co-authors)

Response to Reviewer 1:

1. Line 53: Some mention should be made of studies that suggest California's drought is primarily due to natural variability, such as this recent NOAA report.

We explicitly mention the California drought report (ref. 8) and note its suggestion that the most recent drought is due to natural variability:

Lines 61-64: Climate model simulations forced with observed SSTs alone were unable to capture all the important features of the recent precipitation decline, suggesting that a non-negligible component of California's recent precipitation deficit may be attributable to other factors, such as internal atmospheric variability (8).

2. 61, 66: Hartmann et al 2014 should also be cited here &
3. 67: Kug et al, Nature Geosci. 2015 and Lee et al, GRL 2014 should be cited here.

We have included citations to Hartmann and Ceppi (2014), Kug et al and Lee et al, GRL 2014.

4. 113, Fig. 2a, Fig. S2e, Fig. S6: Changes in absolute precipitation are not terribly meaningful and may obscure regions where substantial fractional changes occur that are small in an absolute sense. I suggest changing the units of these figures to % of mean, which I suspect will illuminate different regions as having substantial changes.

Relative precipitation changes are shown in Fig. 2 and Fig. S9; and we refer to relative precipitation changes in our main text:

Lines 776-780: This is evident from Fig. S9a, which shows that low sea-ice conditions increase the likelihood of a drier California, but do not mean a drier California every year – some years become drier and some wetter. On average, when considering the 20-year mean, this results in a 10-15% precipitation decrease over California (Fig. 2c).

5. 191-192: The intensification of the CA drought since 2013 coincides with the phase shift of the Pacific Decadal Oscillation from negative to positive, as well as the appearance of "The Blob" of warm SSTs off the west coast of North America. The possible influences of these factors should be discussed in the paper.

We now also include a brief discussion of the possible factors contributing to the most recent California drought:

Lines 261-270: This consistency does not, however, constitute compelling evidence that the 2012-2016 California drought is attributable to Arctic sea-ice changes. Rather, it illustrates that some of the atmospheric features of the droughts driven by Arctic sea-ice loss may resemble those of the most recent California drought. The intensification of dry conditions over California since late 2012 may have also been affected by several other factors not discussed in this study, such as the appearance of a large warm SST anomaly off the west coast of North America - though this appears to be a consequence rather than a cause of the altered atmospheric circulations causing the drought (41). Another important factor may be the 2014 phase shift of the Pacific Decadal Oscillation from negative to positive. However, this shift would be expected to alleviate drought conditions over California - not intensify them (42).

6. I think it would be clearer if "low ice," which refers to the Arctic, were termed "low Arctic ice" to differentiate it from "low Antarctic ice." In sections of the text where both are discussed, it is somewhat confusing.

We have changed "low ice" to "low Arctic ice" throughout the manuscript.

7. How does sea-ice thickness change in the low-ice model simulations? What is its influence relative to changes in ice extent?

We thank the reviewer for highlighting this important point. While our experimental design does not allow to quantify the relative influence of sea ice thickness vs extent changes, it is important to clarify that the response described in our study originates from both sea-ice thickness and extent changes. We have clarified this in our Methods and in Supplementary Material Section 1.1 (and at any place in the main text where it may sound that the response is due to area changes only):

Line 401: Analyzed response represents cumulative effect of sea-ice extent and thickness changes.

Lines 688-692: It is important to clarify that the imposed parameter changes do not only affect the sea-ice area, but also the sea-ice thickness. Similar to the sea-ice area changes, thickness changes are also larger in “low Antarctic ice” than in the “low Arctic ice” simulations (not shown). The studied response is of course a consequence of both of these factors – sea-ice thickness and area changes.

8. 216: It’s not clear to me which SST anomalies are being discussed here. Typo: tropic => tropics.

This discussion was an attempt to make reference to the opposite pattern of surface warming compared to the one described in Fig. S2a. We have simplified this sentence to focus on the key features only:

Lines 221-223: Antarctic sea-ice decline will result in a warming at mid- to high-latitudes of the southern hemisphere. One consequence of this warming is a southward shift of tropical precipitation, with convection increase in the southern and central tropical Pacific (40).

9. 241-267: I hope one of the other reviewers has expertise that can assess this comparison with conditions in the distant past. I am not qualified, but it seems that many of the statements are surprisingly certain.

The changes we describe featuring D/O warmings, Bolling/Allerod transition and the Younger Dryas are compiled from a wide range of different proxies and studies. They indicate that the linkages between high- and low-latitude climate changes in the Arctic, tropics, and the U.S. southwest are in very good agreement with the simulation results presented in our paper. The paleoclimatic records from the western US do not have a sufficient resolution to demonstrate the causation, but the highest quality records available (cited in our study) confirm that the changes happening are linked as described by our mechanism.
We emphasize, however, that the changes occurring during the Little Ice Age (LIA) and Medieval Climate Anomaly (MCA) are subject to considerable uncertainty, especially in terms of the past sea ice extent reconstructions. During these periods, therefore, it is more difficult to be confident that covariance relationships between the high- and low-latitude changes are primarily due to the teleconnection described in our study is. We have clarified these issues in our revised manuscript.

Line 294-311: The timing, magnitude, and drivers of sea-ice changes during the last millennium are less well-understood (55). In addition, volcanic activity may have also had an impact on tropical precipitation during this time (56). However, proxy data for the last millennium does yield information regarding the second teleconnection step – the link between the ITCZ location and southwestern rainfall. The southward shift of the ITCZ in the transition from the Medieval Climate Anomaly (MCA) to the Little Ice Age

(LIA) is well-documented (57, 58). The effect of this shift is apparent in the contrast between the dry MCA and the wet LIA in the western US lake levels (59), from trees growing on lake beds exposed by low lake levels (60, 61), and in pollen records (62). Interestingly, a brief southward shift in the ITCZ position between ~AD 1050 and 1250 (57) is clearly recorded in the American southwest as a wet interval in the midst of the dry MCA. Great Basin lakes expanded briefly during this period (63, 64), concurrent with drowning of trees growing below modern water levels (60, 61) and increased soil moisture in southern California (as inferred from pollen records) (62). Such periods during the MCA and LIA provide some support for the second teleconnection step. In the absence of more reliable sea-ice data sets, however, we cannot confirm if such covariance relationships between the ITCZ location and southwestern rainfall are directly attributable to sea-ice changes, or could instead be due to other factors (e.g., volcanic forcing or solar irradiance changes).

10. In figures where statistically significant regions are designated with stippling, the stippling is very difficult to see. I suggest that areas where changes are NOT significant be stippled instead, thereby allowing the stipples to be bolder without obscuring important details.

We agree with the reviewer that the stippling is not as obvious. However, we are also concerned that it may be potentially confusing if we stipple the non-significant regions because it is far less common to use stippling for regions where changes are non-significant. As a compromise solution, we have modified the type of stippling that identifies statistically significant regions. We hope this is a fair compromise. It allows the stippling to be made more obvious without obscuring underlying details of the changes.

11. Fig. 1: Is it correct that the Antarctic "low ice" is for September? I would think that the comparable month would be March, the end of the southern hemisphere summer.

We have changed Fig. 1 to show March instead of September sea ice extent for the 'Antarctic low ice' experiment.

12. Fig. S4: Caption does not identify plots in c and d. Are these figures for DJF?

We thank the reviewer for catching this. We have added this information to the figure caption.

13. Fig. S9b: Changes in 500 hPa heights over the Arctic (decrease) are very different from those shown in Fig. 2b. Any idea why?

We have included fully coupled experiments in order to demonstrate that the observed teleconnection is not an artifact of the AGCM/slab ocean setup, but is also present in a similar (energy-conserving) AOGCM setup. As we note in the revised text, however, the fully coupled setup used (which is described in Cvijanovic et al. 2015), was designed for the specific purpose of investigating the impacts of ocean albedo alteration and does not present a perfect comparison. A number of different factors may explain why there are

certain differences between the response patterns in the AOGCM and the AGCM/slab ocean experimental configurations. These include the way that the sea ice changes are introduced – ocean brightening over an extremely large area in the Arctic (albedo set to 0.7-0.9!) as well as different GHG concentrations. While both of these experimental configurations capture the described 2-step teleconnection, there is indeed a different response poleward of ~70°N. At present, we are unable to assign the feature noted by the Reviewer (the difference in the geopotential changes over Arctic in the AOGCM and AGCM/slab ocean configurations) to one specific factor. We suspect, however, that this difference may be due to increased latent heat transport arising from higher CO_2 concentrations in the fully coupled simulation.

We provide more discussion of this question Section 1.6 of the SM.

Reviewer #2 (Remarks to the Author):

In this contribution, Ivana Cvijanovic and her co-authors use simulations with an AGCM to establish a possible link between sea-ice changes in high latitudes and precipitation changes over California. In doing so, they in particular examine the physical mechanisms that might explain the linkages they find.

I very much enjoyed reading this paper, and found the results presented here both novel and convincing. This work will certainly inspire future studies and is thus in my opinion well suitable for publication in Nature Communications, subject to some rather minor changes as indicated below. I should note that I was particularly impressed by the authors' own capability to discuss the strengths and limits of their approach and to very clearly spell out possible uncertainties in their findings. Because of this, I don't have any substantial criticism regarding the scientific arguments brought forward here.

Minor comments:
l.55: Include possibly reference to Fig. S8b.

We have clarified that all panels are based on European Center for Medium-Range Weather Forecast ERA-Interim reanalysis.

l.77: To me, the term "climate model" today usually refers to an AOGCM. I got confused when I later realised that this term is here meant to represent an AGCM, and think this should be made clear upfront to avoid such confusion.

We have changed the sentence in question to only describe the approach (we describe the AGCM model later in the paragraph):

Lines 80-82: A novel feature of our approach is that we sample the uncertainties in selected sea-ice physics parameters (see Methods and ref. (21)), thereby obtaining an ensemble of simulations with a seasonally ice-free Arctic (‘low Arctic ice’ simulations).

Line 94: We examine this initial response using an Atmospheric General Circulation Model (AGCM) coupled to a mixed-layer (slab) ocean model.

l.80: I wasn't sure if the location of reference (18) is as intended. Doesn't this reference explain the simulations described in the previous sentence, rather than the comparison to the control simulation?

The reviewer is correct, this reference relates to the previous sentence and we have moved it accordingly.

l.87ff: It never became clear to me why this study is not based on the results from the AOGCM that the authors refer to occasionally. While I appreciate that the results might not change qualitatively as discussed in S1.6, the robustness of the results would have been strengthened by using an AOGCM as opposed to an AGCM.

The fully coupled AOGCM experiments were introduced with the aim of demonstrating that the observed teleconnection is not purely an artifact of the AGCM/slab ocean setup, but is also present in a similar (energy-conserving) fully coupled setup. However, the fully coupled AOGCM setup (described in Cvijanovic et al. 2015), was designed for the specific purpose of investigating the impacts of ocean albedo alteration. The AOGCM experiment involves ocean brightening over a large area of the Arctic, in combination with relatively high GHG concentrations. Although the AOGCM experimental configuration is also energy-conserving, it has certain shortcomings. For example, the sea ice changes have been achieved by setting the ocean albedo north of a certain latitude to a high value throughout the entire year. The main value of the AGCM/slab ocean setup is that the sea-ice loss was achieved more directly (via modification of uncertain sea-ice parameters), and without any change in GHG forcing. Further development of a fully-coupled AOGCM setup that utilizes sea-ice physics parameter perturbations will hopefully be possible in the future, but is beyond the scope of our paper.

l.111: It would be nice to show SST also in Fig 2.

Surface temperature changes (SST and TS) have been added to Fig. 2.

l.111-114: I found these remarks distracting within this paragraph. Can they be moved to the Methods?

We have removed the sentence “The winter months are also the time when California receives most of its precipitation”.

l.114-116: Can be removed, as this was already described in l.111.

We have done so.

l.130ff: I found Supplemental Section 1.2 very helpful in understanding these findings. It

would be good if at least some parts of the Supplemental Section could be incorporated into the main text, given its importance to understand the following reasoning.

Thank you. Our primary concern was the manuscript length, but based on the Reviewer's comment we have moved some of Supplementary Section material 1.2 into the main text.

Lines 134-140:
Sea-ice loss leads to a decrease in the net upward TOA shortwave flux that is only partly compensated by an increase in net TOA upward longwave flux. This yields an increased net TOA heat flux into the atmospheric column, and thus less radiation to space (Fig. S3c and f). Compared to the net TOA flux changes, net surface flux changes are relatively small (see Fig. S3a). This is a consequence of the fact that sea-ice induced decrease in the net upward shortwave flux at the surface is almost fully compensated by an increase in the latent and sensible heat fluxes (not shown).

Lines 145-149:
As a consequence of the increased high-latitude heat flux into the atmospheric column, the atmospheric heat transport from mid-latitudes into the high northern latitudes decreases (see the large white arrow in Fig. S4c). The high-latitude energy surplus is compensated for at lower latitudes, with most of the energy emitted to space through the TOA flux changes over the tropical Pacific between 20°S and 20°N (Fig. S4b).

l.241ff: I found the logical flow of the discussion not always clear, as it currently jumps several times between a comparison to observational data and possible limits of the present approach. Instead, one could start with a comparison to recent observational data (l.274-290), the final sentence of which could then nicely be followed by the paragraph in lines 268-273. Then, one could compare the present findings to paleo data, moving back in time. (i.e., first ll 255-267, then ll. 241 to 254). This would then end the comparison to earlier data. Then, the discussion starting in l. 291 could follow.

We have now reorganized the discussion section. We start with a comparison to the most recent drought, then discuss the paleodata, and finally explore the question of whether our results would substantially modified by use of a fully coupled AOGCM. We have also included more detail on other possible causes of the most recent drought. Additionally, we now note that we cannot confirm with high confidence that precipitation changes during the MCA and LIA were triggered by changes in sea-ice. The only suggestion of the Reviewer which we did not implement was to alter the order of discussing the paleoclimatic results. In the submitted version of our manuscript, it may have been unclear that while the 'older' periods appear to be in good agreement with our proposed two-step teleconnection mechanism, the more recent MCA and LIA periods only allow us to discuss the second teleconnection step. This is because the sea-ice changes during the MCA and LIA periods are much less certain. We have clarified this issue further in our revised manuscript. Please see the Discussion Section, lines 246-335.

l.402: Some publications have strange additional notes in capital letters (e.g. 6, 10, 24, 61)

This has been fixed.

Figure 1: I recommend to replace Figure 1b with Figure S1e, and to change the caption accordingly. I'm not sure that it is necessary to include Fig. 1b and S1f. Instead, it can simply be stated that sea-ice coverage in the unperturbed hemisphere is very similar to the control simulation.

We changed Fig. 1 to show March instead of September sea ice extents for the ‘Antarctic low ice’ experiment set (Fig 1b -> Fig. S1e) and excluded Fig. S1f.

Text S1.6 and S1.7: The numbers for references in these two sections seem to be too low by 1. (e.g, ref. (26) should be (27) etc.)

This was corrected.

Fig. S3e: The main text seems to imply that the dashed lines describe changes in clouds. However, I believe that this is not explained in the caption.

We thank the reviewer for noticing this. An explanation has been added to the Figure caption.
